# The Uptake, Trafficking, and Biodistribution of *Bacteroides thetaiotaomicron* Generated Outer Membrane Vesicles

**DOI:** 10.3389/fmicb.2020.00057

**Published:** 2020-02-06

**Authors:** Emily J. Jones, Catherine Booth, Sonia Fonseca, Aimee Parker, Kathryn Cross, Ariadna Miquel-Clopés, Isabelle Hautefort, Ulrike Mayer, Tom Wileman, Régis Stentz, Simon R. Carding

**Affiliations:** ^1^Gut Microbes and Health Research Programme, Quadram Institute Bioscience, Norwich, United Kingdom; ^2^Core Science Resources, Quadram Institute Bioscience, Norwich, United Kingdom; ^3^Earlham Institute, Norwich, United Kingdom; ^4^Biomedical Research Centre, University of East Anglia, Norwich, United Kingdom; ^5^Norwich Medical School, University of East Anglia, Norwich, United Kingdom

**Keywords:** *Bacteroides thetaiotaomicron*, outer membrane vesicles, microvesicles, bacterial extracellular vesicles, gut microbiota, GI-tract, biodistribution, organoid monolayer

## Abstract

Gram-negative bacteria ubiquitously produce and release nano-size, non-replicative outer membrane vesicles (OMVs). In the gastrointestinal (GI-) tract, OMVs generated by members of the intestinal microbiota are believed to contribute to maintaining the intestinal microbial ecosystem and mediating bacteria–host interactions, including the delivery of bacterial effector molecules to host cells to modulate their physiology. Bacterial OMVs have also been found in the bloodstream although their origin and fate are unclear. Here we have investigated the interactions between OMVs produced by the major human gut commensal bacterium, *Bacteroides thetaiotaomicron* (Bt), with cells of the GI-tract. Using a combination of *in vitro* culture systems including intestinal epithelial organoids and *in vivo* imaging we show that intestinal epithelial cells principally acquire Bt OMVs via dynamin-dependent endocytosis followed by intracellular trafficking to LAMP-1 expressing endo-lysosomal vesicles and co-localization with the perinuclear membrane. We observed that Bt OMVs can also transmigrate through epithelial cells via a paracellular route with *in vivo* imaging demonstrating that within hours of oral administration Bt OMVs can be detected in systemic tissues and in particular, the liver. Our findings raise the intriguing possibility that OMVs may act as a long-distance microbiota–host communication system.

## Introduction

The mammalian GI-tract is home to a vast number of microbes that make up the intestinal microbiota which has co-evolved with the host to establish a mutualistic relationship (Round and Mazmanian, 2009). Complex interactions between the intestinal microbiota and the host intestinal epithelium and underlying immune cells play a vital role in maintaining GI homeostasis, host health, and preventing infection (Sommer and Backhed, 2013). Although the mucus layer that coats the entirety of the intestinal epithelium prevents direct contact of luminal microbes with host cells, bacterial products such as metabolites or bacterial OMVs, can access and cross the epithelial barrier (Fateh et al., 2015) to influence both local and systemic host responses (Bomberger et al., 2009; Stentz et al., 2014).

Gram-negative bacteria ubiquitously shed bilayer OMVs into their external environment (Brown et al., 2015; Schwechheimer and Kuehn, 2015). These non-replicative spherical vesicles bud from the bacterial outer membrane and range in size from 20 to 400 nm (Toyofuku et al., 2019). The protective outer lipid bilayer encapsulates and protects their cargo of bioactive proteins, nucleic acids, and metabolites (Bryant et al., 2017). OMVs are increasingly being recognized as a key mode of interkingdom communication between bacteria and host tissues, contributing to a diverse range of functions including nutrient uptake, gene transfer, biofilm formation, antimicrobial protection, and transfer of microbial toxins and virulence factors during infection (Kulp and Kuehn, 2010; Jan, 2017). However, the molecular basis and pathways of host-OMV uptake and the fate of host-cell acquired OMVs and their cargo remain elusive (Margolis and Sadovsky, 2019). Bacterial OMVs have been shown to interact with many different mammalian cell types including IECs (Parker et al., 2010; Bielaszewska et al., 2013; Stentz et al., 2014; O’Donoghue et al., 2017), lung epithelial cells (Bauman and Kuehn, 2009), endothelial cells (Kim et al., 2013), and immune cells (Vidakovics et al., 2010; Yoon et al., 2011; Hickey et al., 2015; Vanaja et al., 2016; Deo et al., 2018). Bacterial DNA of potential OMV origin has also been detected in human blood and urine (Yoo et al., 2016; Lee et al., 2017; Park et al., 2017) as well as in body compartments previously thought to be sterile, such as the heart (Svennerholm et al., 2017), suggesting OMVs can reach distant sites from their site of origin and production, including the lumen of the GI-tract (Stentz et al., 2018).

Historically, studies of OMVs have focused on those produced by pathogenic Gram-negative bacteria and their role in transporting virulence factors and toxins into host cells (Kunsmann et al., 2015; Bielaszewska et al., 2017; Deo et al., 2018; Rasti et al., 2018). Recently, studies have emerged showing OMVs released by commensal and probiotic bacteria may confer beneficial effects on the host by maintaining microbial and GI-tract homeostasis by influencing host epithelial and immune cell responses. For example, we have shown that OMVs generated by the major human commensal gut bacterium Bt can activate host immune responses when delivered intranasally and via the GI-tract (Carvalho et al., 2019). Probiotic *Escherichia coli* strain Nissle 1917 OMVs have been shown to aid in maintaining the gut barrier by upregulating expression of barrier enhancing TJ proteins zonula occludens-1 and claudin-14 (Alvarez et al., 2016), and by enhancing production of antimicrobial proteins and anti-inflammatory cytokines (Fabrega et al., 2016, 2017; Alvarez et al., 2019). Similarly, OMVs generated by *Bacteroides fragilis* have been shown to elicit immunomodulatory effects and prevent gut inflammation in a mouse model of colitis (Shen et al., 2012).

Although these findings highlight the ability of OMVs to influence host cell physiology, we still do not fully understand the diverse mechanisms of OMV uptake and cargo delivery. The study of OMV uptake is challenging due to their nano-size and the fact that the molecular mechanisms OMVs might use to drive microbiota–host interactions are poorly understood compared to studies of pathogenic bacteria (Stentz et al., 2018). Several OMV internalization pathways have been identified for certain bacterial species including actin-dependent macropinocytosis, clathrin-mediated endocytosis, caveolin-mediated endocytosis, or clathrin- and caveolin-independent mechanisms such as membrane fusion or lipid raft formation (O’Donoghue and Krachler, 2016). However, uptake of OMVs generated by commensal microbiota species such as *Bacteroides* spp. have not been studied in detail. The aim of the present study therefore was to evaluate Bt OMV uptake and trafficking pathways within host cells and track their biodistribution using the strain VPI- 5482. This strain is widely used as a model commensal bacterium for investigating host–bacteria interactions (Hooper et al., 2003; Eckburg et al., 2005; Rakoff-Nahoum et al., 2014; Stentz et al., 2014, 2015; Zakharzhevskaya et al., 2017). Using a combination of *in vitro* and *in vivo* imaging techniques we have shown that commensal Bt OMVs are internalized by IECs via several routes including dynamin-dependent endocytosis, macropinocytosis, and caveolin-mediated endocytosis and are ultimately sorted to a peri-nuclear localization through host–cell endo-lysosomal pathways. We also demonstrate that a proportion of Bt OMVs localize to cellular junctions whereby they can cross the intestinal epithelium by paracellular transmigration to disseminate widely throughout the host.

## Materials and Methods

### Animal Handling

Eight- to twelve-week-old C57BL/6 and Atg16l^ΔIEC^ (Jones et al., 2019) single sex mice were bred and maintained in the University of East Anglia (United Kingdom) animal facility. All mice were housed in individually ventilated cages and exposed to a 12 h light/dark cycle with free access to water and a standard laboratory chow diet. Animal experiments were conducted in full accordance with the Animal Scientific Procedures Act 1986 under UK HO approval and HO project license 70/8232.

### Mammalian Cell Culture

The human colonic epithelial cell line Caco-2 (ECACC 86010202) was cultured at 37°C and 5% CO_2_ in Dulbecco’s Modified Eagle Medium with 4.5 g/L glucose and 2 mM L-glutamine (Sigma) supplemented with 5% fetal bovine serum (Lonza), 1% non-essential amino acids (Sigma), penicillin (100 U/ml), and streptomycin (100 μg/ml) (Sigma).

### Intestinal Organoid Monolayer Culture

Small intestinal or caecal crypts were isolated from mouse tissue using a modified method of Jones et al. (2019). Briefly, the GI-tract tissues were opened longitudinally, washed in ice-cold DPBS then cut into 5-mm pieces. The tissue fragments were incubated in GCDR (StemCell Technologies) for 15 min then transferred to ice-cold DPBS for shaking, then returned to GCDR for 5 min. This process was repeated until three to five fractions were generated and then inspected for released crypts. The crypt suspensions were passed through a 70-μm filter to remove debris, then centrifuged at 300 × *g* for 5 min. Crypt pellets were resuspended in murine organoid growth media (StemCell Technologies) supplemented with 10 μg/ml rho-associated coiled-coil containing protein kinase inhibitor (Y-27632, TOCRIS) and seeded onto culture ware coated with Cultrex reduced growth factor basement membrane matrix, type 2 (R&D Systems) at a density of 1000 crypts/ml.

### Bacterial Strains and OMV Isolation

Bt VPI-5482 was grown under anaerobic conditions at 37°C in BHI medium (Oxoid) supplemented with 15 μM hemin or with 0.75 μM hemin for OMV preparations. Bt OMVs were isolated and purified following a method adapted from Stentz et al. (2014). Briefly, cultures (500 mL) of Bt were centrifuged at 5500 × *g* for 45 min at 4°C and the supernatants filtered through 0.22-μm pore-size polyethersulfone membranes (Sartorius) to remove debris and cells. The supernatants were concentrated by crossflow ultrafiltration (100 kDa MWCO, Vivaflow 50R, Sartorius) and the retentate rinsed once with 500 mL of PBS (pH 7.4). The OMV suspensions were concentrated to 1 ml in sterile PBS, filtered through 0.22 μm pore-size syringe-filters (Sartorius), and stored at 4°C. The sterility of the OMV suspension was confirmed by plating onto BHI–hemin agar. The size and concentration of Bt OMVs was determined using a Nanosight nanoparticle instrument (Malvern Instruments). A 1-min AVI file was recorded and analyzed using nanoparticle tracking analysis (Version 2.3 Build 0011 RC, Nanosight) software to calculate size distributions and vesicle concentrations, expressed as particle size (nm) versus number of particles per milliliter. The settings were as follows: calibration: 166 nm/pixel; blur: auto; detection threshold: 10, minimum track length: auto, temperature: 21.9°C, and viscosity: 0.96 cP.

### Fluorescence Microscopy

Bt OMVs (1 × 10^11^/mL) were labeled with 5% (v/v) DiO or DiD Vybrant cell-labeling solution (Molecular probes) by incubating at 37°C for 30 min. Unbound dye was removed by washing with 3× PBS using centrifugal filters (100 kDa MWCO, Sartorius). Labeled OMVs (1 × 10^10^/mL) were added to Caco-2 monolayers cultured on collagen solution (Sigma) coated 24-well chamber slides (IBIDI) or primary mouse organoid monolayers cultured on BME2-coated slides for up to 48 h. This OMV concentration was determined to represent optimal fluorescence signal for microscopy imaging (data not shown). Samples were fixed using Pierce 4% PFA (ThermoFisher), permeabilized with 0.25% Triton X100 (Sigma), and blocked with 10% goat serum in PBS. Intracellular membranes were visualized using Alexa 647-Phalloidin, anti-PDI for ER, anti-58k for Golgi network, anti-Tomm20 for mitochondria, anti-Lamin B1 for nuclear membrane, anti-Rab5 for early endosomes, anti-LAMP1 for lysosomes, and anti-E-cadherin for lateral cell membranes. All secondary antibodies were Alexa 594-conjugated goat anti-rabbit or anti-mouse unless otherwise stated. Antibodies were prepared using TBS (50 mM) (pH 7.6; Sigma) as diluent containing 1% bovine serum albumin (Sigma). For nuclear visualization, cells were incubated with Hoechst 33342 (ThermoFisher). TBS was used as a wash buffer throughout (unless otherwise stated) and all incubations were carried out at ambient temperature. Cells were mounted with high precision glass slides (IBIDI) using Fluoromount-G antifade mounting medium (SouthernBiotech). Images were taken using a Zeiss Axioimager.M2 microscope, equipped with a Plan-Apochromat 63×/1.4 oil immersion objective and ZEN blue software. Fluorescence was recorded at 405 (blue, nucleus), 488 (green, OMVs), and 594 nm (red, Alexa-594 immunostaining). Uptake of DiO-OMVs by Caco-2 was quantified using sum fluorescent pixel intensity of the field of view using a macro written in Image J/FIJI v1.52p. The arbitrary fluorescence units were normalized to PBS control and the mean of each group expressed as average fluorescence intensity (AU). For pH-dependent staining of lysosomes, Caco-2 were cultured on collagen-coated glass coverslips in a 24-well plate prior to treatment with Bt OMVs as above and staining with LysoID red detection kit (ENZO) according to manufacturer’s instructions. Images were taken as above with an EC Plan-Neofluar 20×/0.50 objective. All image analysis was performed using Image J/FIJI v1.52p. Yellow coloration in merged image panels indicates co-localization of Alexa-594 immunostaining with DiO-OMVs. Co-localization analysis of pixel intensity was quantified using the Coloc2 plugin in Image J/FIJI v1.52p and represented in the text as Pearson’s correlation coefficient (*r*). In addition, a Zeiss LSM880 Airyscan confocal microscope equipped with a 63×/1.4 Oil DIC objective and ZEN black software (ZEISS) was used to obtain higher resolution images with *Z*-stack images (6.4–7.6 μm) at 0.38–0.4 μm per slice.

### Live Imaging Using Confocal Fluorescence Microscopy

Caco-2 monolayers were cultured on collagen (Sigma) coated 35 mm glass bottom μ-dishes (IBIDI) and stained with CellTracker red CMTPX dye (ThermoFisher) according to manufacturer’s instructions. Briefly, cells were incubated with 5 μM CellTracker red CMTPX dye for 15 min followed by repeated washes with cell media. Cells were treated with DiO-labeled Bt OMVs (1 × 10^10^/mL) and live imaging immediately performed using a Zeiss LSM880 Airyscan confocal microscope equipped with a W N-Achroplan 63×/0.9 dipping objective. Fluorescence was recorded at 405 (blue, nucleus), 488 (green, OMVs), and 602 nm (red, CellTracker). *Z*-stacks at 0.9 μm per slice were acquired using ZEN Black software (ZEISS). All image analysis was performed using Image J/FIJI v1.52p. Yellow coloration in merged image panels indicates co-localization of CellTracker with DiO-OMVs.

### Electron Microscopy

Bt OMVs were observed using negative staining with transmission electron microscopy (TEM) as previously described (Stentz et al., 2015). Briefly, isolated Bt OMVs were adsorbed to carbon–formvar-coated copper EM grids (Agar Scientific) for 1 min before wicking off with filter paper and negatively staining with 2% uranyl acetate solution (BDH) in water for 1 min. Grids were air-dried before analysis using a Tecnai G2 20 Twin TEM (FEI) at 29,000× magnification.

### Endocytosis Assay

Caco-2 monolayers were pre-treated with inhibitors of endocytosis: Dynasore (80 μM; clathrin- and caveolin-mediated endocytosis inhibitor), cytochalasin D (1 μg/mL; macropinocytosis membrane fusion inhibitor), chlorpromazine (15 μg/mL; clathrin-dependent endocytosis inhibitor), nystatin (50 μM; caveolin-mediated endocytosis and lipid raft inhibitor), or amiloride (10 mM; macropinocytosis inhibitor) (all Sigma) for 1 h at 37°C and 5% CO_2_ with gentle rocking prior to treatment with Bt OMVs (1 × 10^10^/mL) or PBS control for 6 h. Samples were washed with 3 × PBS and extracellular DiO fluorescence quenched using 0.025% trypan blue (Sigma) prior to fixing with Pierce 4% PFA (ThermoFisher) for 20 min. OMV uptake was quantified by fluorescence microscopy using a Zeiss Axioimager.M2 equipped with an EC Plan-Neofluar 40×/0.75 objective and ZEN blue software. Fluorescence was recorded at 488 nm (green, OMVs) and image analysis was performed using Image J/FIJI v1.52p. Internalization of DiO-OMVs was quantified using sum fluorescent pixel intensity of the field of view using a macro written in Image J/FIJI v1.52p. The mean arbitrary fluorescence units of each group were expressed as % of control.

### Transepithelial Electrical Resistance

Transepithelial electrical resistance measurements were performed using a 24-well plate Transwell system (Greiner). Caco-2 monolayers were seeded on the apical compartment of 0.4 μm transparent polyethylene terephthalate (PET) membrane inserts until fully confluent. Bt OMVs (1 × 10^10^/mL) or PBS control were added to the apical compartment and TEER measurements recorded using an EVOM^2^ epithelial voltmeter with chopstick electrode (World Precision Instruments Inc.).

### FITC–Dextran Translocation

Using the Transwell system above, confluent Caco-2 monolayers were treated with 1 mg/mL 3–5 kDa FITC–dextran (Sigma) in the media of the apical compartment. Translocation of fluorescent FITC–dextran into the basal media compartment was recorded using a FLUOStar OPTIMA (BMG Labtech) at excitation 485 and 520 nm emission.

### *In vivo* Biodistribution Imaging

Eight-week-old female C57BL/6 mice (*n* = 4/grp) were orally administered DiD-labeled Bt OMVs at a dose of 2 × 10^10^ OMV/mouse or PBS (200 μL total volume/mouse). Organs including heart, lungs, liver, kidney, spleen, and entire GI-tract were excised at 8 h post administration and far-red fluorescence acquired using an *in vivo* Xtreme multi-modal optical and x-ray small animal imaging system (Bruker) equipped with a back-illuminated 4MP CCD detector. Foreground far-red DiD fluorescence was recorded with the following settings: excitation 650 nm and emission 700 nm, 19 cm field of view, 20 s exposure time, fStop 1.1, and focal plane 0. Background image was recorded by reflectance as above with an exposure time of 1 s. Radiant efficiency of each organ was measured using Bruker Molecular Imaging software (v 7.2.0.21148) by overlaying foreground and background images and recording organs as individual regions of interest (ROI). Data were displayed as absolute fluorescence (photons/s/mm^2^) or normalized to PBS control and calculating % fluorescence of all organs.

### Statistical Analysis

All data are presented as the mean ± standard error of the mean (SEM) with the indicated sample sizes. Data were subjected to D’Agostino and Pearson omnibus normality test and *p*-values calculated using Student’s unpaired *t*-test (fluorescence uptake assay) one-way ANOVA followed by Dunnett’s (endocytosis assay) or Bonferroni *post hoc* tests (OMV uptake assay, FITC–dextran assay, and biodistribution assay) or two-way ANOVA followed by Bonferroni *post hoc* test (TEER assay) using GraphPad Prism 5 software (version 5.04). Statistically significant differences between two mean values were considered when ^∗^*p* = 0.05, ^∗∗^*p* < 0.01, ^∗∗∗^*p* < 0.001, and ^****^p < 0.0001.

## Results

### Characterization of Bt OMVs

**FIGURE 1:**
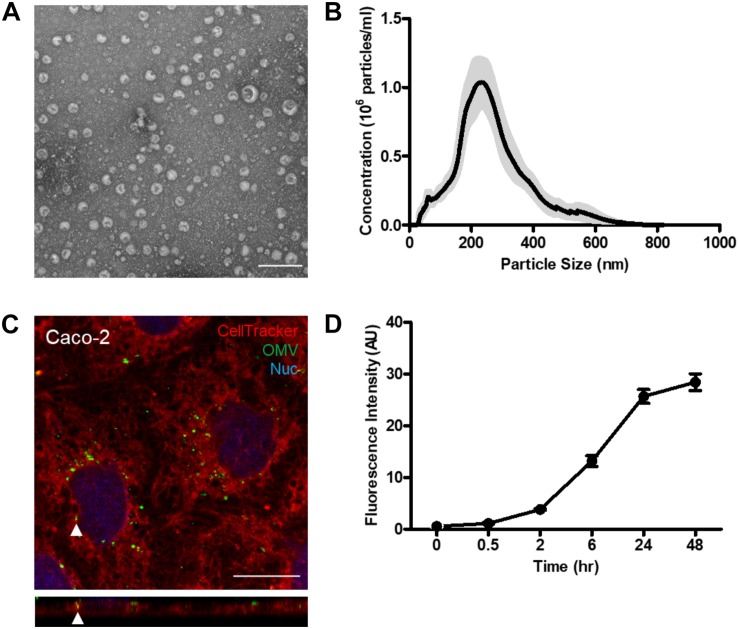
Bt OMVs are rapidly acquired by intestinal epithelial cells. **(A)** Bt generates OMVs of heterogeneous sizes when grown in nutrient-rich media as shown by TEM. Scale bar = 200 nm. Images are representative of more than three independent preparations. **(B)** Size distribution and concentration of Bt OMVs using Nanosight NTA. Data are representative of more than three independent preparations with the gray shading representing the extent of variation of the different preparations. **(C)** Visualizing uptake of DiO-labeled Bt OMVs by Caco-2 monolayers using live imaging and confocal microscopy. Cell Tracker red CMTPX dye was used to visualize Caco-2 cells. The main panel shows merged XY image with the bottom panel showing XZ orthogonal view. Arrow heads indicate intracellular OMVs. The images shown are representative of more than three independent experiments. **(D)** The uptake of DiO-OMVs by Caco-2 monolayers increases over 48 h as determined by quantification of DiO-OMVs using sum fluorescent pixel intensity of each field of view using a macro written in Image J/FIJI v1.52p. The arbitrary fluorescence units were normalized to PBS control samples and the mean of each group expressed as average fluorescence intensity (AU). The graph depicts mean ± SEM values from one independent experiment with ≥10 technical replicates.

Bt generated OMVs were isolated using ultracentrifugation and filtration (Stentz et al., 2014). Electron microscopy imaging showed that the isolated Bt OMV population comprised of spherical, bilayered nano-sized vesicles of various sizes (Figure 1A). Dynamic light scattering revealed Bt OMVs ranged in size from 20 to 400 nm with a mean size of approximately 200 nm (Figure 1B). Furthermore, the majority (62.0%) were routinely between 100 and 200 nm, with 40.8% between 200 and 400 nm, with 0.5% <100 nm, and with 0.46% >400 nm (data not shown).

### Bt OMV Uptake by Intestinal Epithelial Cells

Using live cell imaging, it was observed that within 15 min of exposure to Caco-2 cells Bt OMVs were associated with the apical cell membrane with some already being internalized and in close proximity to the nucleus (Figure 1C). To determine the kinetics of OMV uptake, DiO-labeled Bt OMVs were incubated with Caco-2 monolayers and intracellular fluorescence intensity quantified at various timepoints over a 48 h period. Fluorescence from extracellular OMVs was quenched using trypan blue. A small proportion of Bt OMVs were internalized after 30 min (1.4%), which increased over time up to 24 h at which time a plateau of OMV uptake was evident (35.6%). A significant proportion of OMVs were still visible 48 h after administration (39.4%) (Figure 1D and Supplementary Figure S3) which was not statistically different from the 24 h timepoint (*p* = 0.197). Therefore, for subsequent studies of OMV uptake and intracellular trafficking, the 24 h timepoint of incubation was used. To confirm the intracellular fluorescence was attributable to Bt OMVs and not free dye, OMV–Caco-2 co-cultures were stained with an in-house generated rabbit anti-Bt OmpA (BT_3852) antisera. DiO OMVs co-localized with OmpA immune-labeled OMVs, confirming the DiO signal was specifically associated with Bt OMVs (Supplementary Figure S1A). Over the time course of the imaging study, Caco-2 cell viability was unaltered (Supplementary Figure S1B).

### Intestinal Epithelial Cells Internalize Bt OMVs Primarily via Dynamin-Dependent Endocytosis

**FIGURE 2:**
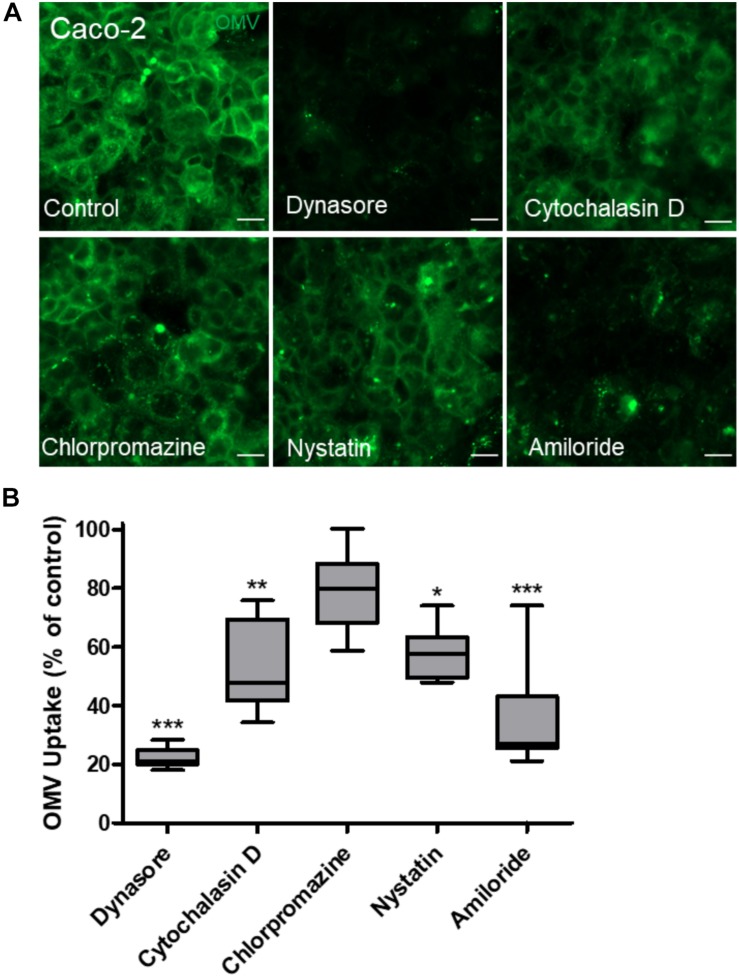
Dynamin-dependent endocytosis is the main route by which Bt OMVs are acquired by intestinal epithelial cells. **(A)** Caco-2 monolayers were incubated with the endocytosis inhibitors Dynasore, cytochalasin D, chlorpromazine, nystatin, or amiloride for 1 h prior to the addition of DiO-OMVs which were subsequently visualized by fluorescence microscopy. The images shown are representative of those obtained from two independent experiments. Scale bars = 20 μm. **(B)** Internalized DiO-OMVs were quantified using sum fluorescent pixel intensity of each field of view using a macro written in Image J/FIJI v1.52p. The arbitrary fluorescence units were normalized to PBS control samples and the mean of each group expressed as average fluorescence intensity (AU). The data are depicted as the mean of each group expressed as % of control. The box plots depict mean ± SEM of ≥10 images and the whiskers min–max values. *p < 0.05, **p < 0.01, ***p < 0.001.

The route of bacterial OMV entry by host cells can occur via several endocytosis pathways, often with multiple pathways being utilized concurrently (Bielaszewska et al., 2013, 2017; Olofsson et al., 2014; Kunsmann et al., 2015; Canas et al., 2016). Therefore, to determine the mechanism of Bt OMV uptake by IECs, chemical inhibitors were used to block specific endocytosis pathways using published protocols (Bielaszewska et al., 2013; Kunsmann et al., 2015). DiO-labeled Bt OMVs were incubated with Caco-2 monolayers for 24 h prior to trypan blue quenching and quantification of internalized Bt OMVs by fluorescence microscopy (Figure 2A). All inhibitors reduced Bt OMV uptake compared to non-treated control with dynamin-dependent Dynasore and macropinocytosis inhibitor amiloride significantly reducing OMV uptake (by 78.1 and 63.42%, respectively). By comparison, caveolin-mediated inhibitor nystatin (42.32%) and macropinocytosis F-actin inhibitor cytochalasin D (47.85%) had only a moderate effect, with clathrin-mediated inhibitor chlorpromazine (21.11%) having the lowest reduction in OMV uptake (Figure 2B). These results indicate that uptake of Bt OMVs occurs predominantly via dynamin-dependent endocytosis or macropinocytosis with caveolin- and clathrin-mediated routes of endocytosis also being used but to a lesser degree.

### Intracellular Trafficking of Bt OMVs

**FIGURE 3:**
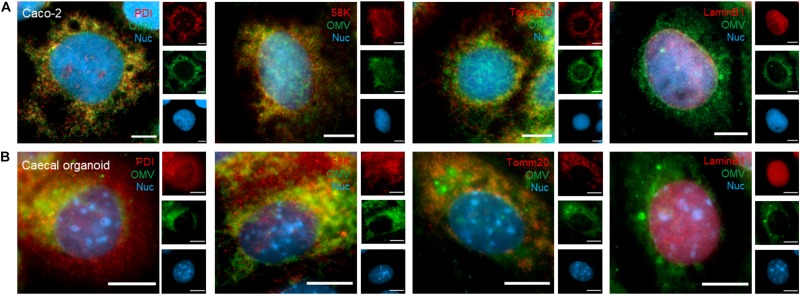
Intracellular trafficking of Bt OMVs. **(A)** Caco-2 monolayers and **(B)** caecal organoid epithelial monolayers were incubated with DiO-OMVs for 24 h after which samples were stained with antibodies to visualize the ER (PDI), Golgi network (58K), mitochondria (Tomm20), nuclear membrane (Lamin B1), and the nucleus (Hoechst 33342). Stained cells were imaged by fluorescence microscopy. The main panels represent merged images from three separate channels that are individually shown in the side panels. Images are representative of more than three independent experiments. Scale bars = 20 μm.

As internalized Bt OMVs consistently localized to an intracellular compartment, we sought to identify the pathway(s) of Bt OMV trafficking within IECs. To visualize OMV association with host–cell organelles, DiO-labeled Bt OMVs were incubated with Caco-2 monolayers or murine caecal- or SI-derived organoid monolayers for 24 h and assessed by immunofluorescence microscopy. Organoid cell monolayers were used to reflect the heterogeneous populations of absorptive and secretory cell types within the intestinal epithelium and to provide a comparison with the widely used Caco-2 model. Both caecal and SI-derived IECs form polarized monolayers within 16 h with apical microvilli and TJ that comprised the major cell types of the caecal- and SI epithelium *in vivo* as determined using antibodies specific for stem cells, goblet cells, and enteroendocrine cells (Supplementary Figures S2A–C). Co-localization of Bt OMVs with host cellular organelles was investigated using the ER marker PDI, the Golgi apparatus marker 58k, the mitochondrial marker Tomm20, and the nuclear envelope marker Lamin-B1. In Caco-2 cells, co-localization (denoted by yellow coloration in merged image panels of Figure 3) was observed between DiO-OMVs and the Golgi apparatus (*r* = 0.599), ER (*r* = 0.573), and mitochondria (*r* = 0.554) (Figure 3A). In caecal organoids (Figure 3B) co-localization was observed between DiO-OMVs and Golgi apparatus (*r* = 0.422), ER (*r* = 0.417), and mitochondria (*r* = 0.516). This suggests that after endocytic uptake at the apical membrane Bt OMVs traffic to the host Golgi apparatus, ER, and mitochondria. Although direct DiO-OMV co-localization with the nuclear envelope was not quantifiable in Caco-2 (*r* = 0.003) or caecal organoid monolayers (*r* = 0.037), DiO-OMVs were observed in very close proximity with the nucleus at a distance of between 0.00 and 11.23 μm with a median distance of 0.819 μm for Caco-2 and between 0.00 and 7.782 μm with a median distance of 0.800 μm for caecal organoids.

**FIGURE 4:**
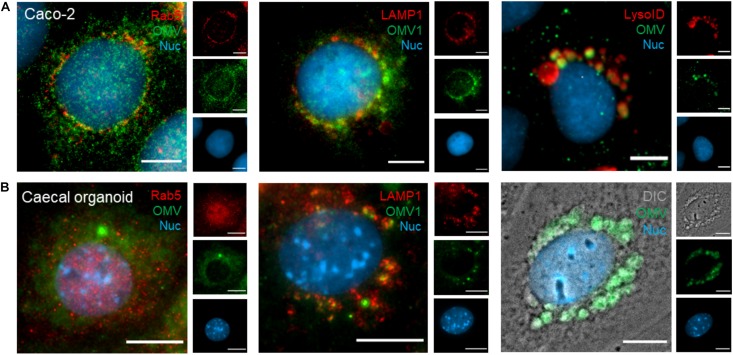
Endo-lysosomal trafficking of Bt OMVs. **(A)** Caco-2 monolayers and **(B)** caecal organoid epithelial monolayers were treated with DiO-OMVs for 24 h, fixed and stained with antibodies to visualize early endosomes (Rab5), lysosomes (LAMP1 and LysoID), and the nucleus (Hoechst 33342). The main panels depict merged images with the side panels showing the individual channels. Images are representative of more than three independent experiments. Scale bars = 20 μm.

**FIGURE 5:**
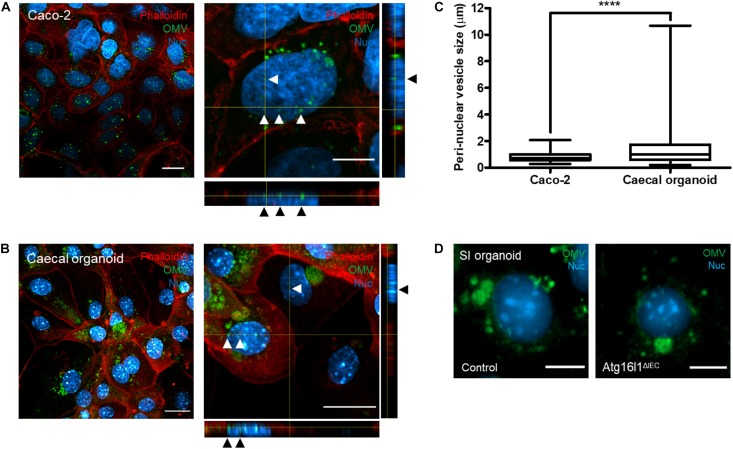
Localization of Bt OMVs to a peri-nuclear membrane. **(A)** Caco-2 cell monolayers and **(B)** caecal organoid epithelial monolayers were incubated with DiO-OMVs for 24 h and stained with phalloidin to visualize the apical membrane and the nuclear stain Hoechst 33342 prior to imaging by confocal microscopy. The main panels depict merged XY images with the side panels showing XZ and XY orthogonal views. Arrow heads indicate peri-nuclear localization of OMVs. Images are representative of more than three independent experiments. Scale bars = 20 μm. **(C)** Quantification of the host peri-nuclear vesicle size was performed using the straight-line selection tool in Image J/FIJI v1.52p. The box plots depict mean ± SEM and the whiskers min–max values from more than three independent experiments with more than 200 vesicles quantified per group. **(D)** Primary SI epithelial organoid monolayers were generated from wild-type control (Left) or autophagy-deficient (Atg16l1^ΔIEC^) mice (Right) and incubated with DiO-OMVs as above and analyzed by fluorescence microscopy. The main panels depict merged images with the side panels showing the individual channels. Images are representative of more than three independent experiments. Scale bars = 20 μm. ****p < 0.0001.

The peri-nuclear accumulation of DiO-positive host vesicles suggested some OMVs may be sequestered by the host endo-lysosomal pathway. To identify peri-nuclear vesicles, DiO-labeled OMVs were incubated with Caco-2 monolayers and caecal organoid monolayers for 24 h and OMV co-localization with early endosomes (Rab5^+^) and lysosomes (LAMP1^+^ and LysoID^+^) investigated (denoted by yellow coloration in merged image panels of Figure 4). A small population of peri-nuclear OMVs was shown to co-localize with Rab5^+^ early-endosomes in Caco-2 cells (*r* = 0.371) but not caecal organoid monolayers (*r* = 0.166) (Figures 4A,B). Peri-nuclear OMVs were shown to co-localize with LAMP1/LysoID in Caco-2 cells (*r* = 0.325 and *r* = 0.727 respectively) and with LAMP1 in caecal monolayers (*r* = 0.581) (Figures 4A,B), suggesting the peri-nuclear vesicles containing OMVs are late endosomes or lysosomes. Additionally, since LysoID detects only organelles with an acidic pH, Bt OMV uptake did not inhibit endo-lysosomal acidification. Clear association between Bt OMVs and large DiO-positive host-derived peri-nuclear vesicles was seen in both caecal and SI organoid monolayers (Figure 4B and data not shown). This co-localization of OMVs with the peri-nuclear membrane was confirmed using high-resolution confocal microscopy, in which DiO-positive OMVs were shown to be in intimate association with the nucleus in both Caco-2 and caecal organoid monolayers (Figures 5A,B). Interestingly, the vesicles presented as frequent, small puncta (0.2–2.1 μm) in Caco-2 monolayers but as larger host-associated vesicular compartments (0.2–10.7 μm) in organoid monolayers (Figure 5C). Taken together these results indicate that Bt OMVs traffic to the host Golgi apparatus and ER as well as the nucleus via the endo-lysosomal pathway where they ultimately accumulate in lysosomal vesicles, presumably for membrane fusion and release of their cargo.

### Intracellular Trafficking of Bt OMVs Is Autophagy Independent

To establish if OMV trafficking to lysosomes via the endo-lysosomal pathway is autophagy dependent, an IEC specific, Atg16l1-deficient mouse model (Atg16l1^ΔIEC^) was used as previously described (Jones et al., 2019). The Atg16l1 protein is a key component of the canonical autophagy pathway and alongside proteins such as LC3 is required for formation of autophagosomes. Consequently, Atg16l1^ΔIEC^ mice show reduced autophagy (Jones et al., 2019). Using SI organoid monolayers generated from wild-type and Atg16l1^ΔIEC^ mice the localization of DiO-labeled Bt OMVs to peri-nuclear host-associated vesicles was comparable in both WT and Atg16l1^ΔIEC^ organoid monolayers (*p* = 0.074) (Figure 5D) excluding any role of autophagy as a cellular process in Bt OMV intraepithelial trafficking.

### Bt OMV Transmigration of the Intestinal Epithelium

**FIGURE 6:**
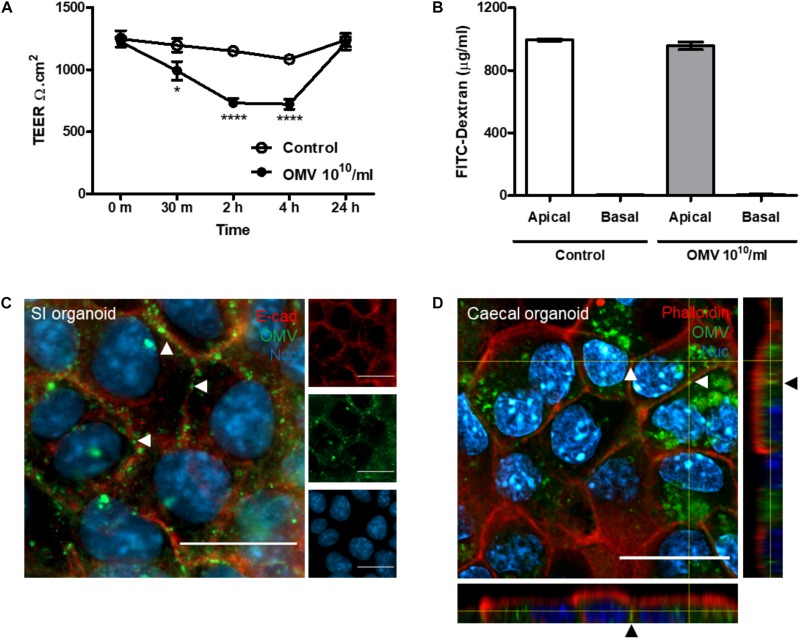
Bt OMV transmigrate the host GI-tract. **(A)** Non-labeled Bt OMVs were administered to the apical compartment of tissue culture inserts containing a confluent, polarized monolayer of Caco-2 cells. Transepithelial electrical resistance (TEER) was measured at regular intervals post-treatment. The graph depicts mean ± SEM values from two independent experiments with five technical replicates. **(B)** To assess cell permeability, 3–5 kDa FITC-labeled dextran (1 μg/ml) was added to the apical compartment during OMV administration and FITC fluorescence measured in both the apical and basal compartments at 24 h post-administration of OMVs. The bar graph depicts mean ± SEM values from two independent experiments with five technical replicates. **(C)** Small intestinal (SI) organoid epithelial monolayers were incubated with DiO-OMVs for 24 h and stained with antibodies to visualize lateral cell membranes (E-cadherin) and with a nuclear stain (Hoechst 33342) prior to fluorescence microscopy. The main panel shows merged images with the side panels depicting the separate channels. Arrow heads indicate lateral cell membrane localization of OMVs. **(D)** Caecal organoid epithelial monolayers were incubated with DiO-OMVs for 24 h and stained with phalloidin to visualize apical cell membranes and the nuclear stain Hoechst 33342 prior to analysis by confocal microscopy. The main panels show merged XY images and the side panels show XZ and XY orthogonal views. Arrow heads indicate paracellular localization of OMVs. Images are representative of more than three independent experiments. Scale bars = 20 μm. *p < 0.05, ****p <0.0001.

In addition to cellular uptake, OMVs can transmigrate across the host intestinal epithelium and reach the lamina propria to access underlying immune cells, the vasculature, and systemic tissues (Stentz et al., 2018). Caco-2 monolayers were used to determine if Bt OMVs alter the integrity of the IEC barrier using a cell culture insert system to measure TEER and translocation of 3–5 kDa FITC-labeled dextran. In comparison with PBS control, a significant reduction in TEER was observed within 2 h of OMV administration which was restored by 24 h (Figure 6A). In contrast, there was no evidence of FITC–dextran translocation in response to Bt OMV exposure (Figure 6B) and no loss or redistribution of the TJ proteins ZO-1 and occludin were observed Supplementary Figure S4), suggesting Bt OMVs can modulate certain aspects of the intestinal epithelial barrier.

To investigate this further, fluorescence and confocal imaging was used to visualize Bt OMV interaction with cellular junctions and their transmigration across epithelial cell monolayers. After 24 h exposure, Bt DiO-OMVs were seen to localize to lateral cellular membranes of murine SI organoid monolayers, forming a distinct chicken-wire like pattern of OMV puncta (Figure 6C). Confocal imaging of caecal organoid monolayers revealed that DiO-OMVs were found between cells in a basolateral location (Figure 6D). Collectively this data suggest Bt OMVs transiently modulate the host TJ barrier in order to transmigrate across the intestinal epithelium via the paracellular pathway.

### *In vivo* Biodistribution of Bt OMVs Following Oral Administration

**FIGURE 7:**
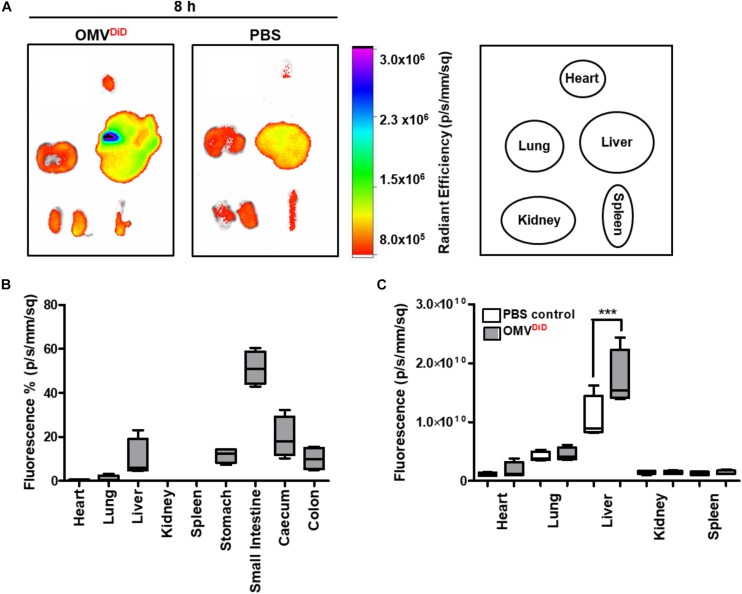
*In vivo* biodistribution of Bt OMVs following oral administration. **(A)** Mice were orally administered with DiD-OMVs (OMV^DiD^, 2 × 10^10^/mouse) or PBS and individual organs (Right) excised at 8 h post-administration for imaging using a Bruker *in vivo* Xtreme imaging system. The images shown are representative of those obtained from four mice per group. **(B,C)** The graphs depict the proportion **(B)** or absolute **(C)** amount of the fluorescence signal from the OMV^DiD^ inoculum or PBS control administered to each animal that was subsequently detected in each organ. The box plots depict mean ± SEM and the whiskers min–max values. ***p < 0.001.

Following our observation that Bt OMVs transmigrate across the intestinal epithelium, we sought to determine if after oral administration Bt OMVs could reach systemic tissues. Using an *in vivo* model adapted from those previously described (Park et al., 2010, 2018; Jang et al., 2015; Wiklander et al., 2015; Kim et al., 2017), far-red fluorescent DiD-labeled Bt OMVs were orally administered to mice for 8 h prior to organ excision and imaging using a Bruker *in vivo* Xtreme imaging system. This time-point of tissue collection was optimal to observe the early stages of Bt OMV biodistribution to tissues via the blood stream. Radiant efficiency of each organ was normalized to PBS control. An increase in far-red fluorescence was observed in various organs of DiD-OMV treated mice compared to PBS control (background autofluorescence) (Figure 7A). The highest signal was detected in the GI-tract and in particular the SI (51.35%), stomach (11.71%), caecum (19.70%), and colon (10.14%). Lower intensity signals were evident in systemic tissues including the liver (9.99%), lungs (1.04%), and heart (0.63%) (Figure 7B). When the far-red fluorescence of each organ was analyzed separately to the tissues of the GI-tract, only the liver exhibited a significant increase in DiD fluorescence (Figure 7C). As a whole, our *in vivo* biodistribution data suggest that Bt OMVs can translocate through the host GI-tract to reach various systemic tissues with the greatest accumulation in the liver.

## Discussion

Bacterial OMVs normally produced by Gram-negative bacteria are increasingly being recognized as a secretory inter- and intra-kingdom communication system (Jan, 2017; Cecil et al., 2019). However, many features of this mechanism remain to be defined (Margolis and Sadovsky, 2019). Due to their nano-size and biophysical properties, OMVs have the potential to cross the sterile mucous layer that coats the intestinal epithelium to gain access to host cells and in particular, boundary epithelial cells. However, their nano-size also proves a challenge in identifying their specific routes of uptake by and trafficking within host cells. Here we have exploited lipophilic dyes that are highly fluorescent upon incorporation into the OMV membrane bilayer (Parker et al., 2010; Bielaszewska et al., 2013; Mulcahy et al., 2014; Kunsmann et al., 2015) to visualize Bt OMVs and track their uptake and intracellular localization in cultured IECs of the caecum and small and large intestine, and their subsequent biodistribution *in vivo*.

Using high-resolution live-imaging we demonstrate that Bt OMV cellular uptake occurs rapidly (within 15 min) in IEC monolayers, which is consistent with previous studies (Furuta et al., 2009b; Parker et al., 2010; Olofsson et al., 2014; Kunsmann et al., 2015). In contrast to studies using pathogen-derived OMVs that are generally toxic (Furuta et al., 2009a; Bielaszewska et al., 2017), Bt OMVs have no discernable adverse effect on epithelial viability. Uptake of Bt OMVs by IECs occurs via all four main pathways of endocytosis: actin-driven macropinocytosis, clathrin-mediated endocytosis, caveolin-mediated endocytosis, or non-caveolin- and non-clathrin-mediated endocytosis (O’Donoghue and Krachler, 2016) with dynamin-dependent endocytosis and macropinocytosis being the most prominent. The utilization of different pathways by Bt OMVs for their uptake may reflect their size heterogeneity (20 to >400 nm) and the size selectivity of each route of endocytosis with macropinocytosis for instance allowing uptake of OMVs < 1 μm whereas clathrin- and caveolin-mediated endocytosis enables uptake of smaller OMVs < 120 nm (O’Donoghue and Krachler, 2016). This interpretation is supported by the recent finding that the size of *Helicobacter pylori* OMVs determined their mechanism of endocytosis (Turner et al., 2018).

**FIGURE 8:**
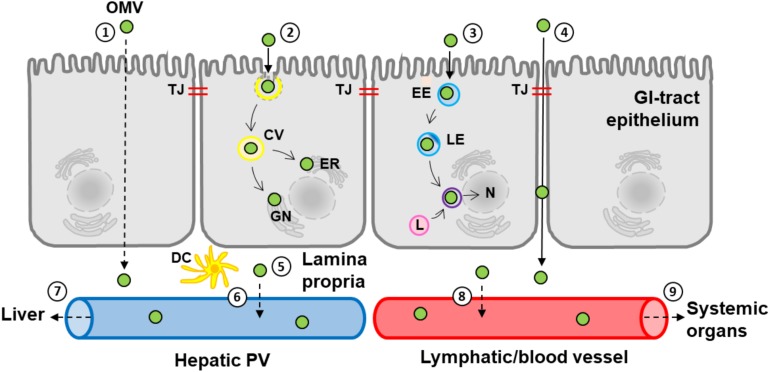
A schematic representation of the uptake and fate of Bt OMVs in the GI-tract. The diagram depicts the predicted pathways of Bt OMV uptake and transmigration of the host GI-tract epithelium to reach the systemic circulation and systemic organs such as the liver. (1) Transcellular transmigration. (2) Caveolin-mediated endocytosis and subsequent inclusion in caveolar vesicles (CV) and trafficking to the endoplasmic reticulum (ER) and Golgi network (GN). (3) Endo-lysosomal trafficking via early endosomes (EE), late endosomes (LE), and lysosomes (L) to co-localize to the nucleus (N) and the peri-nuclear membrane. (4) Paracellular transmigration and passage through tight junctions (TJ). (5) Translocation to lamina propria and interaction with immune cells such as dendritic cells (DCs). (6) Distribution to the hepatic circulation and portal vein (PV) and subsequent delivery to the liver (7). (8) Distribution to lymphatic vessels and/or the systemic circulation and dissemination to more distant organs such as the heart and lungs (9). The solid arrows identify confirmed pathways with dashed arrows representing predicted pathways.

Most endocytic routes of Bt OMV uptake culminate in lysosomes located in a peri-nuclear region (Furuta et al., 2009b; Bielaszewska et al., 2013; Figure 8), which we found does not require autophagosome formation and therefore autophagy (Tooze et al., 2014). We have observed that intracellular Bt OMVs persist within lysosmes for >72 h (data not shown) suggesting they may resist intracellular degradation for prolonged periods of time after their acquisition. As the caveolin-dependent endocytosis pathway bypasses lysosomes it is likely that OMVs are transported via the caveolar network to the host ER/Golgi apparatus (Figure 8) thereby escaping lysosomal degradation (Kiss and Botos, 2009; Figure 8). An important caveat to this interpretation is that endocytosis pathways used by transformed, proliferating cells in culture such as Caco-2 may differ to those in primary cells such as the intestinal epithelium where quiescent, senescent, and terminally differentiated cells are present (Hinze and Boucrot, 2018). Our use of primary epithelial cells in the form of organoid monolayers which reflect the cellular heterogeneity of the intestinal epithelium *in vivo* represents one approach to overcoming the limitations of using immortalized cell lines.

Our findings reveal that commensal Bt OMVs are internalized by primary murine organoid monolayers derived from the SI and caecum which contain proliferating and non-proliferating cell types such as goblet cells and enteroendocrine cells (Sato et al., 2009; Zachos et al., 2016). Some differences in the fate of Bt OMVs between organoid and Caco-2 cell culture systems were evident. Larger peri-nuclear host-associated vesicles, of up to 10.7 μm, were observed in primary organoid monolayers compared to smaller peri-nuclear host-associated vesicle puncta (average 0.8 μm) in Caco-2 cells. This may reflect differences in lysosomal storage, degradation, and exocytosis of Bt OMVs between immortalized and tumor-derived Caco-2 cells and primary IECs. Further studies are required to determine how these differences relate to the fate of internalized OMVs and their potential delivery to the nucleus.

Under normal healthy conditions, the selectively permeable TJ barrier of the intestinal epithelium allows the flux of ionic solutes (leak pathway) as well as larger non-charged molecules (pore pathway) (Shen et al., 2011). As paracellularly located OMVs were only observed in primary caecal organoid cultures and not Caco-2 monolayers, this may reflect different TJ pore capacities of the different cell types. TJs in primary cells are “leakier” than in Caco-2 monolayers (Van Itallie et al., 2008; Turner et al., 2014) as reflected by electrical resistance (TEER) and permeability (FITC–dextran) measurements demonstrating that only the leak pathway appears to be modulated by Bt OMVs. It was therefore not surprising that OMVs could not be detected in basal supernatants of Caco-2 monolayers (data not shown). The more permeable TJ barrier of primary compared to immortalized IECs enables the paracellular transmigration of a small population of Bt OMVs (Figure 8), allowing them to access underlying cells and the vasculature (Figure 8). A disrupted TJ barrier as a result of injury or inflammation could lead to a greater proportion of Bt OMVs being translocated across the GI-tract to host tissues. Further investigations are required to determine if Bt OMV cellular uptake and fate changes in disease states.

Based on our *in vitro* findings and the fact that bacterial EVs have previously been detected in human serum (Tulkens et al., 2018), we speculated that orally administered Bt OMVs can cross the murine GI-tract and be delivered to other tissues systemically. We have shown that within 8 h of oral administration small numbers of labeled OMVs are evident in several organs and most notably the liver. In contrast to the parenteral administration of OMVs described in previous studies (Jang et al., 2015; Wiklander et al., 2015; Kim et al., 2017; Park et al., 2018), oral administration of Bt OMVs facilitates their interaction with the host GI-tract that is analogous to that of endogenously produced OMVs (Sonnenburg et al., 2005; Porter et al., 2017). The finding that the vast majority of Bt OMVs remain in the lumen of the GI-tract suggests that the general fate of OMVs generated *in vivo* is excretion. Our biodistribution study does however indicate that a small population of luminal Bt OMVs can enter the circulatory or lymphatic systems via the GI-tract. As the liver was the primary site for OMV biodistribution in our study, it is likely that OMVs transmigrating through the intestinal epithelium enter the hepatobiliary system (Figure 8). The finding of Bt OMVs in the heart and lungs also suggests that some OMVs can also enter the blood stream. As OMVs are proteoliposomes, they likely move through the lymphatics prior to entering the blood circulation (Figure 8). Our results suggest therefore that Bt OMVs interact with and can cross several host cellular barriers including the intestinal epithelial barrier and the lymphatic- and vascular-endothelium (Figure 8).

While our combined *in vitro* and *in vivo* approach to studying OMV-mediated interactions between members of the intestinal microbiota with their host has verified the delivery of intact Bt OMVs into and across the intestinal epithelium, identifying the pathways of Bt OMV transport within the body and the sites of delivery of their cargo remains a challenge.

## Conclusion

We have shown that OMVs from the prominent gut commensal bacterium Bt have the potential to act as a long-distance microbiota–host communication system. Bt OMVs interact with cells of the GI-tract via several endocytosis pathways, ultimately localizing to endo-lysosomal vesicles in close proximity to the peri-nuclear membrane. Additionally, a proportion of Bt OMVs transmigrate through epithelial cells via a paracellular route and *in vivo* can cross the IEC barrier to reach systemic organs. Understanding in more detail the biodistribution pathways and ultimate targets of OMVs is key to elucidating their benefit to host health.

## Data Availability Statement

All datasets generated for this study are included in the article/Supplementary Material.

## Ethics Statement

The animal experiments were conducted in full accordance with the Animal Scientific Procedures Act 1986 under the UK HO approval and HO project license 70/8232.

## Author Contributions

SC, EJ, and RS conceived and designed the experiments. EJ and SC wrote the manuscript. SC supervised the work. EJ, CB, SF, AP, KC, RS, AM-C, and IH executed the experimental work. EJ carried out the data interpretation and statistical analysis. UM and TW provided the reagents and/or advice. All authors revised, and read and approved the final manuscript.

## Conflict of Interest

The authors declare that the research was conducted in the absence of any commercial or financial relationships that could be construed as a potential conflict of interest.

## Abbreviations

BHI: brain heart infusion
Bt: *Bacteroides thetaiotaomicron*
DiD: 1,1 ′ -dioctadecyl-3,3,3′,3′ -tetramethylindocarbocyanine perchlorate
DiO: 3,3-dioctadecyloxacarbocyanine perchlorate
ER: endoplasmic reticulum
EV: extracellular vesicle
FITC: fluorescein isothiocyanate
GCDR: gentle cell dissociation reagent
GI: gastrointestinal
HO: Home Office
IEC: intestinal epithelial cell
OmpA: outer membrane protein-A
OMV: outer membrane vesicle
PFA: paraformaldehyde
SI: small intestine
TBS: tris buffered saline
TEER: transepithelial electrical resistance
TEM: transmission electron microscope
TJ: tight junction.
