# Supplementary material for: The Uptake, Trafficking, and Biodistribution of Bacteroides thetaiotaomicron Generated Outer Membrane Vesicles
Source: Front Microbiol. 2020 Feb 6;11:57. doi: 10.3389/fmicb.2020.00057 (PMC7015872; doi:10.3389/fmicb.2020.00057)
Supplement: Supplementary file 1 [file Data_Sheet_1.pdf]

Supplementary Material

Materials and Methods

Fluorescence microscopy

Bt OMVs (1x10^{11}/ml) were labelled with 5% 3,3 dioctadecyloxacarbocyanine perchlorate (DiO) or 1,1'-dioctadecyl-3, 3, 3', 3'-tetramethylindocarbocyanine perchlorate (DiD) Vybrant cell-labelling solution (Molecular probes, USA) by incubating at 37°C for 30 minutes. Unbound dye was removed by washing with 3 x PBS using centrifugal filters (100 kDa MWCO, Sartorius). Labelled OMVs (1x10^{10}/ml) were added to Caco-2 monolayers or isolated primary mouse crypts cultured on collagen solution (Sigma) or BME2 coated 24-well chamber slides (IBIDI, Germany) for up to 24hrs. Samples were fixed using Pierce 4% paraformaldehyde (PFA; ThermoFisher), permeabilized with 0.25% Triton X100 (Sigma) and blocked with 10% goat serum in PBS. Bt OMVs were visualized according to the DiO fluorescence or using affinity-purified antibodies raised against major outer membrane protein OmpA [Bt_3852] (Carvalho et al. 2019) followed by Alexa 594 conjugated goat anti-mouse IgG (ThermoFisher). Cell types in murine SI and caecal organoid monolayers were visualized using antibodies specific for stem cells (Sox9), goblet cells (Muc2) and enteroendocrine cells (ChrA). Tight junction (TJ)-associated proteins were visualized using antibodies specific for zonula occludens-1 (ZO-1) or occludin. All secondary antibodies were Alexa 594-conjugated goat anti-rabbit or anti-mouse IgG (thermoFisher). Tight junction (TJ) associated proteins were visualized using antibodies specific for zonula occludens-1 (ZO-1) or occludin. Antibodies were prepared using Tris (50mM) buffered saline (TBS, pH 7.6; Sigma) as diluent containing 1% bovine serum albumin (BSA; Sigma). For nuclear visualization, cells were incubated with Hoechst 33342 (ThermoFisher, UK). TBS was used as a wash buffer throughout and all incubations were carried out at ambient temperature. Cells were mounted with glass slides (IBIDI) using Fluoromount-G antifade mounting medium (SouthernBiotech). Images were taken using a Zeiss Axioimager.M2 microscope, equipped with a Plan-Apochromat 63x/1.4 oil immersion objective and ZEN blue software. Fluorescence was recorded at 405nm (blue, nucleus), 488nm (green, OMV) and red (Alexa 594, OmpA).

Cell Viability

Labelled Bt OMVs (1x10^{10}/ml) were added to confluent Caco-2 monolayers cultured in 24-well tissue culture plates for up to 24hrs. Caco-2 monolayer viability was assessed by incubating cells post-exposure to OMVs with resazurin (20%) and measuring fluorescence 1 hr later at excitation 550nm and 584nm emission with a FLUOStar OPTIMA (BMG Labtech).

Electron Microscopy

For transmission electron microscopy (TEM), murine small intestinal (SI) organoid monolayers were cultured on phenol-red free, reduced growth factor Matrigel (Corning) coated Thermanox coverslips (ThermoFisher) and fixed with Pierce 4% PFA (ThermoFisher). Following fixation, samples were washed 3 x PBS and dehydrated through an alcohol gradient followed by resin embedding in LR white resin (Agar Scientific). Samples were sectioned at 90nm and collected onto formvar/carbon coated copper EM grids (Agar Scientific). Grids were stained sequentially with 2% uranyl acetate for 1 hr at ambient temperature and 0.5% lead citrate-tribasic trihydrate (Sigma) for 1 min at ambient temperature, with 5 x deionized water

washes between steps. Grids were dried before imaging using a Tecnai20 TEM (FEI) at 2500x magnification.

Figures

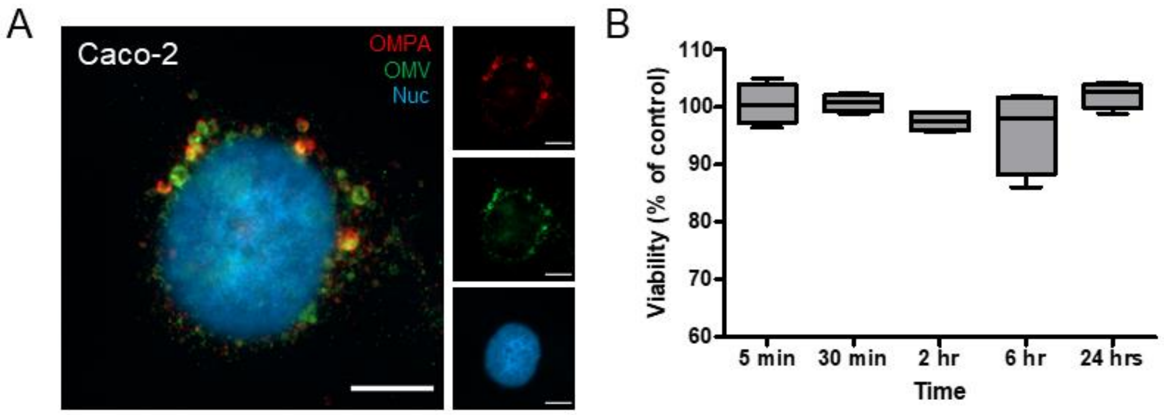

Figure S1: Identification of Bt OMVs in IECs and their impact on cell viability A) To confirm that DiO fluorescent signal from DiO-labelled Bt OMVs detected by fluorescence microscopy is specific for Bt OMVs, Caco-2 monolayers exposed to DiO-OMVs for 24hr, were fixed and stained with an anti-Bt OmpA (OMPA) antibody and Hoechst 33342 nuclear stain prior to fluorescence microscopy. The main panel shows a merged image of co-localized DiO and OMPA antibody fluorescence (yellow) with the side panels showing separate channels. The images are representative of more than three independent experiments. Scale bar = 20µm. (B) Caco-2 viability was assessed at regular intervals post-OMV treatment by incubating cell monolayers with resazurin (20%) for 1 hr and measuring production of fluorescent resorufin in cell media. The values shown represent the mean ±SEM values from one independent experiment with four technical replicates.

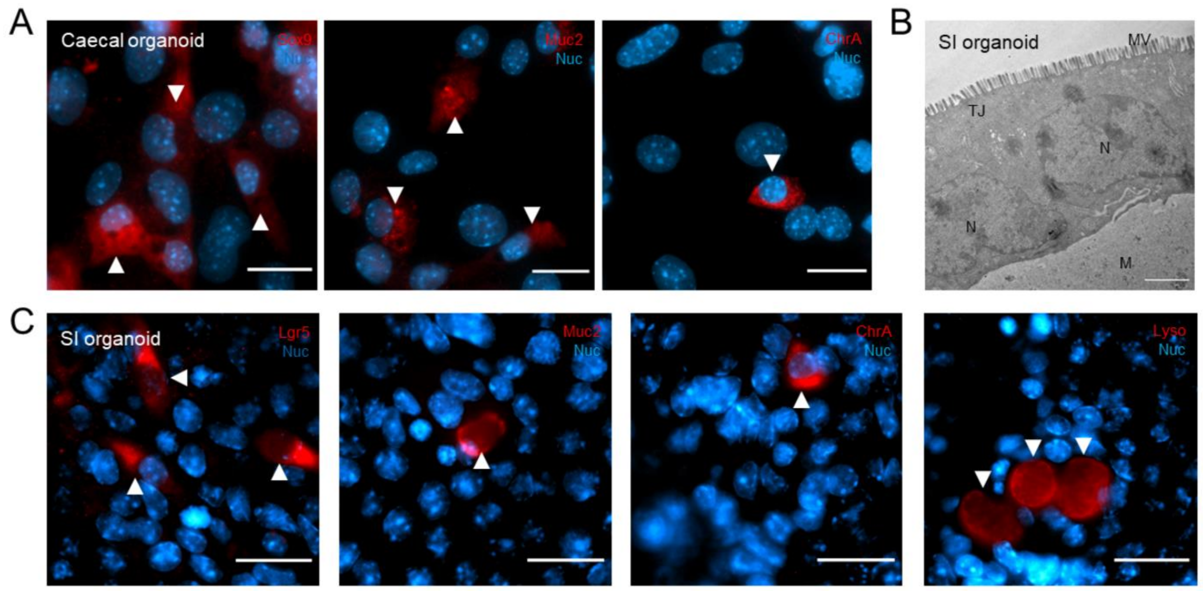

Figure S2: Characterization of murine organoid epithelial monolayer cultures. Caecal (A) and small intestinal (B) organoid monolayers contain the differentiated cell types found in the intestinal epithelium *in vivo*. Organoid monolayers were fixed and stained with antibodies to detect stem cells (Sox9), goblet cells (Muc2), enteroendocrine cells (ChrA), and Paneth cells (Lyso) and with the nuclear dye Hoechst 33342 prior to fluorescence microscopy. The arrow heads identify individual antibody-stained cells. Images are representative of those obtained from at least three independent experiments. Scale bar = 20µm. (B) Ultrastructure of murine small intestine (SI) organoids as visualized by TEM and negative staining. Images are representative of those obtained from three independent samples. Scale bar = 2µm. Abbreviations: MV, microvilli; TJ, tight junction; N, nucleus; M, Matrigel.

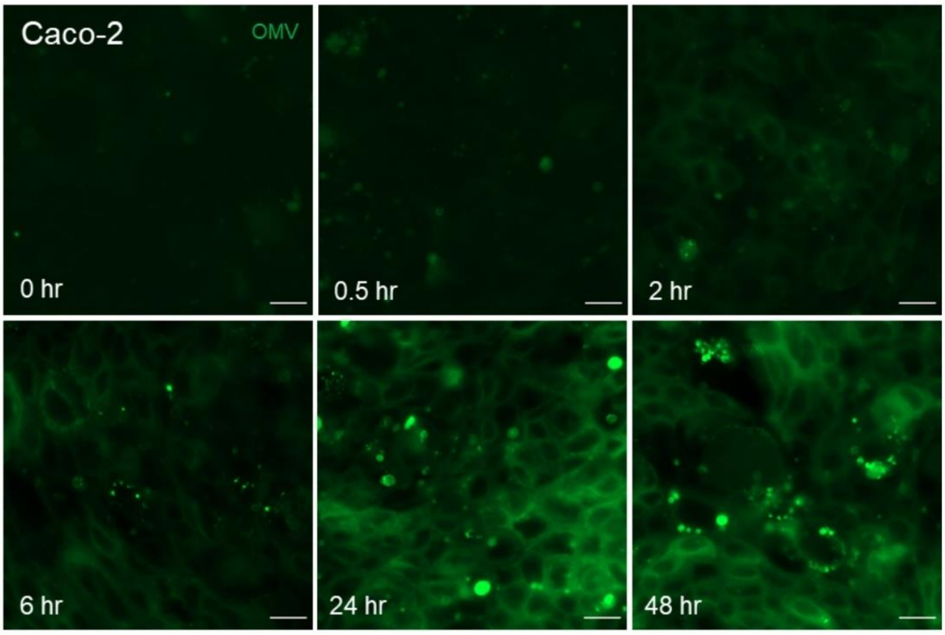

Figure S3: Uptake of Bt OMVs by intestinal epithelial cells. The uptake of DiO-OMVs by Caco-2 monolayers increases over 48 hr. Caco-2 monolayers were incubated with DiO-OMVs for 0-48 hrs then fixed and subsequently visualized by fluorescence microscopy. The images are representative of those obtained from two independent experiments. Scale bars = 20μm.

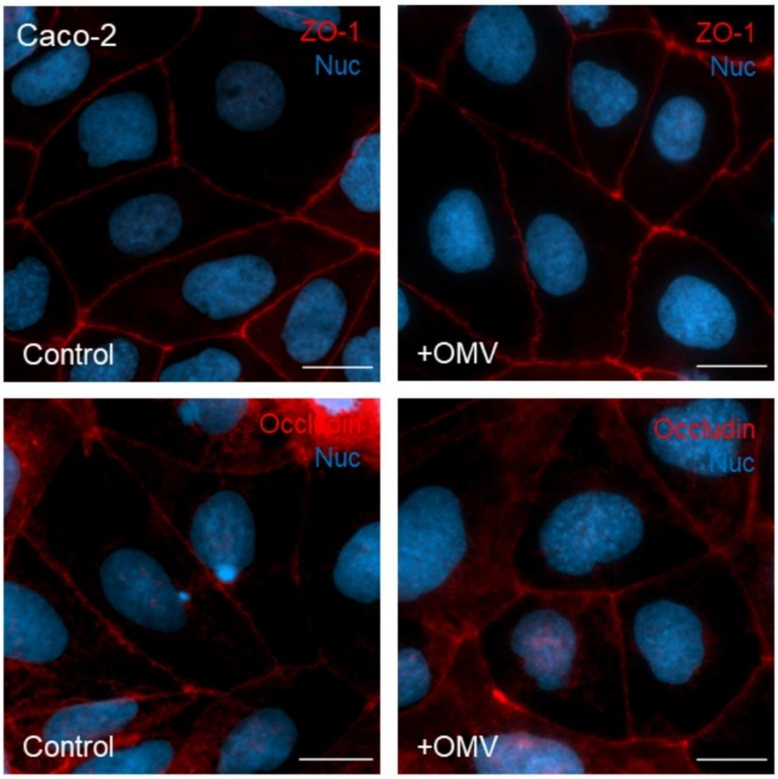

Figure S4: Bt OMV impact on intestinal epithelial tight junction proteins. Caco-2 monolayers were exposed to DiO-OMVs for 24hr then fixed and stained with antibodies specific for tight-junction (TJ)-associated proteins ZO-1 and occludin and nuclear stain Hoechst 33342 prior to fluorescence microscopy. The images are representative of more than three independent experiments. Scale bar = 20µm.
